# Clinical Effectiveness of Neoadjuvant Chemotherapy in Gastric Carcinoma and Exploration of Perioperative Imaging Assessment Parameters

**DOI:** 10.1155/2021/5563136

**Published:** 2021-04-23

**Authors:** Jiajun Lai, Junsheng Li, Xianwei Mo

**Affiliations:** ^1^Department of Gastrointestinal Surgery, Yuebei People's Hospital, Shaoguan, Guangdong, China 512025; ^2^First Department of Surgery, Guilin Hospital of Integrated Traditional Chinese and Western Medicine, Guilin, Guangxi, China 541004; ^3^Department of Gastrointestinal Surgery, Guangxi Cancer Hospital of Guangxi Medical University, Nanning, Guangxi, China

## Abstract

**Background and Aims:**

Due to the difficulty in clinical staging, a simple and feasible perioperative assessment approach for guiding personalized neoadjuvant chemotherapy (NAC) is lacking. We investigated the clinical value of NAC in advanced gastric carcinoma (GC) and the concordance between perioperative imaging and postoperative pathological assessments.

**Methods:**

This study included 62 patients with advanced GC who received NAC between January 2012 and December 2018. The preoperative and postoperative T stages, postoperative pathological tumor regression grade (TRG), and changes in computed tomography (CT) values after NAC were assessed. Follow-ups were conducted to obtain the median survival time (MST), and Kaplan–Meier survival curves were plotted.

**Results:**

The T stages significantly differed between before and after NAC (*p* = 0.001). The MST of patients in the TRG0 group was significantly different from that of patients in the TRG1+2 and TRG3 groups (*p* = 0.223). The percentages of positive lymph nodes were 0%, 24.17%, and 27.64% in the TRG0, TRG1+2, and TRG3 groups, respectively. TRG was correlated with changes in CT values before and after NAC, and the extent of change was associated with patient prognosis.

**Conclusions:**

Perioperative imaging can be used to assess the short-term effectiveness of NAC for patients with GC.

## 1. Introduction

Gastric carcinoma (GC) is a malignant tumor originating from the epithelial lining of the stomach. Approximately 1.2 million new cases of GC are reported annually worldwide, and 40% of these new cases are reported in China. According to the 2020 “World Cancer Report” published by the World Health Organization, GC is the third leading cancer in China in terms of incidence and mortality [1]. In China, early-stage GC accounts for less than 20% of all diagnosed GC cases, and most patients are in the advanced stage of the disease at diagnosis. The current treatment strategy for GC incorporates a multimodal approach that primarily involves surgery. Specifically, radical D2 gastrectomy is the only cure for advanced GC. However, patients often miss the opportunity for surgery due to extensive tumor invasion and metastasis or invasion of adjacent organs, in which case, the only option would be palliative resection. The 5-year overall survival (OS) of patients with advanced GC is less than 50%, and their prognosis is poor [2].

In recent years, gastrointestinal cancer has been considered a systemic disease, which cannot be cured completely with surgery alone. The results of the MAGIC and FFCD9703 trials clearly showed that compared with surgery alone, perioperative chemotherapy could increase the radical resection rate of GC and improve patient prognosis [3]. The concept of neoadjuvant chemotherapy (NAC) was first proposed by Frei et al. in 1982, who showed that NAC improved OS and disease-free survival; their results led to the recommendation of NAC in patients with gastrointestinal cancer [4]. Notably, promising efficacy has been demonstrated in several phase II studies with the safe use of D2 gastrectomy or more extended surgery following NAC [5, 6]. In addition, a meta-analysis showed that patients in the NAC group had higher radical resection rates (odds ratio (OR) = 1.900, *p* < 0.01) and OS (OR = 1.83, *p* < 0.01) than those in the surgery-alone group [7], and the 5-year and 10-year survival rates of patients who received the combination of surgery and adjuvant chemotherapy increased by 5.8% and 7.4%, respectively, when compared with those of patients who received surgery alone. These findings demonstrate that NAC can increase the radical resection rate and improve survival in patients with advanced GC [3, 4, 7, 8].

Studies at multiple cancer treatment centers have demonstrated that NAC is effective in downstaging tumors, lowering the pathological tumor regression grade (TRG), and prolonging patient survival [9–14]. However, due to the difficulty in clinical staging, a simple and feasible perioperative assessment approach that can be used to guide personalized treatment is lacking. Computed tomography (CT) is accurate in terms of target localization and characterization, provides clear anatomical structures, and is convenient to use. Thus, it is the preferred method for the clinical staging of GC [9–14]. In this study, we tried to examine the concordance between perioperative CT examinations and postoperative pathological assessments and explored the potential of perioperative imaging for assessing the short-term effectiveness of NAC for patients with GC.

## 2. Methods

Clinical data of patients with advanced GC who were diagnosed and treated at the Department of Gastrointestinal Surgery of Guangxi Cancer Hospital of Guangxi Medical University between January 2012 and December 2018 were collected. Advanced GC was defined according to the National Comprehensive Cancer Network guidelines in oncology [15]. The exclusion criteria were as follows: presence of serious complications, such as upper gastrointestinal hemorrhage; no tolerance to surgery or presence of distant metastasis; prior antitumor therapy; history of malignant tumor; previous subtotal gastrectomy; missing clinical pathological data; presence of any severe or uncontrollable systemic disease; and breastfeeding or pregnancy. After excluding the patients with the above criteria, the remaining patients with gastric adenocarcinoma, confirmed by gastroscopy and biopsy, were selected for inclusion in this study. In addition, patients had to be 18–80 years old and have an Eastern Cooperative Oncology Group (ECOG) score of 0–2. Finally, a total of 62 patients met these criteria and were included. Contrast-enhanced CT was performed before NAC and surgery. Patients received 3–4 cycles of chemotherapy with the S-1 (Shandong Xin Shi Dai Pharmacy, Qingdao, Shandong, China) and oxaliplatin (Jiangsu Hengrui Medicine, Lianyungang, Jiangsu, China) regimen (S-1 was taken orally at 40 mg/m^2^ from the 1st to 14th days, twice every day, followed by 7 days of rest, plus oxaliplatin infused intravenously at 130 mg/m^2^ on the first day) [15], followed by radical D2 gastrectomy.

A retrospective analysis of patients' clinical data, including sex, age, tumor location and size, depth of invasion of the tumor (T), lymph node status, degree of tissue differentiation, TNM stages before NAC and after surgery, and arterial phase CT values of the tumor tissue before and after NAC, was performed. The basic characteristics of the 62 patients are shown in Table 1. Pathological tumor regression grading was performed after surgery, and the performance status was scored according to the ECOG scale.

Follow-ups were conducted according to the Guidelines for the Diagnosis and Treatment of Gastric Carcinoma developed by the Chinese Medical Association Gastric Carcinoma Expert Committee [2]. Patients were followed up in the hospital and outpatient clinics or via telephone. Follow-ups were conducted every 3–6 months in postoperative years 1 and 2, every 6–12 months in postoperative years 3 and 5, and once a year after postoperative year 5. During the follow-up visits, blood biochemistry tests for tumor marker assessment, gastroscopy, and imaging examinations were performed. The cutoff date of the follow-up period was December 2018.

The collected data were analyzed using the SPSS version 22.0. OS was defined as the time from treatment initiation to death or the last follow-up (for patients who were lost to follow-up). OS rates were compared using the Kaplan–Meier survival analysis. The log-rank test was used to determine whether the survival distribution of patients with different pathological regression grades (TRG0–3) was the same at each time point. A *p* value of <0.05 was considered statistically significant.

## 3. Results

### 3.1. Differences in the TNM Stage between before NAC and after Surgery

The TNM staging developed by the American Joint Committee on Cancer was used to categorize the patients into 9 groups (stages 0–IV). Tumor staging was performed before NAC and after surgery in the 62 enrolled patients. Differences in the tumor stage were analyzed, and the results are shown in Table 2. A retrospective analysis of the relationship between the postoperative TRG and postoperative pathological stage was performed. We first classified the patients based on their TRG, followed by the clinical TNM stage. In the TRG0 group, 6 patients had postoperative pathological stage 0 tumors. In the TRG1+2 groups, 3 patients had postoperative pathological stage I tumors, 12 patients had stage II tumors, and 13 patients had stage III tumors. In the TRG3 group, 5 patients had postoperative pathological stage II tumors, 22 patients had stage III tumors, and 1 patient had stage IV tumors. Results of the nonparametric rank-sum test indicated that changes in the TNM stage of GC from before NAC to after surgery were not statistically significant (*p* = 0.84). Therefore, the TNM staging is not a suitable criterion for determining the effectiveness of NAC.

### 3.2. T Stages before NAC and after Surgery

The postoperative TNM stage was assessed based on the postoperative pathological analysis, which exhibits a high accuracy for T and N stages. Moreover, the preoperative TNM stage was assessed based on the CT examination, which is relatively accurate for the T stage but is less accurate for the N stage. Thus, major errors might be caused when comparing TNM stages based on perioperative imaging for the assessment of NAC effectiveness. Therefore, we sought to compare the T stages before NAC and after surgery.  Table 3 shows the distributions of T stages before NAC and after surgery. The results showed that changes in the T stage from before NAC to after surgery were statistically significant (*p* = 0.001). This finding indicates that NAC could reduce the T stage in some patients with advanced GC.

### 3.3. Differences in the Survival Time across TRG Groups

**Figure 1:**
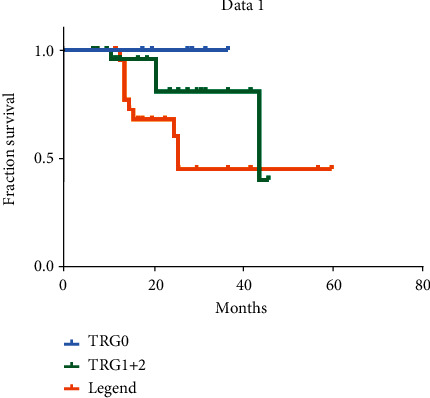
Survival curves of the 62 patients who underwent NAC and radical gastrectomy based on pathological regression grades. The *X*-axis denotes the survival time in months. TRG0 (blue curve) refers to the group with a significant pathological response, TRG1+2 (green curve) refers to the group with a partial pathological response, and TRG3 (orange curve) refers to the group with no pathological response.

Follow-up results in TRG groups showed no death in the TRG0 group, 4 deaths in the TRG1+2 group, and 11 deaths in the TRG3 group. The survival curves of TRG groups are shown in Figure 1. The nonparametric log-rank test was used to analyze the relationships between the curves. The results showed that the TRG0 group (1; blue) had a significantly different prognosis from the TRG1+2 (2; green) or TRG3 groups (3; yellow) (*p* = 0.023) (Figure 1). Meanwhile, the prognosis was not significantly different between the TRG1+2 (2) and TRG3 (3) groups (*p* = 0.11). The median OS rates of patients in the TRG0, TRG1, TRG2, and TRG3 groups were 28.5, 22.5, 16, and 16.5 months, respectively. Since most patients in the TRG3 group had TNM stage III tumors, the median survival time observed in our study is consistent with that of patients with stage III tumors in a similar study [16].

### 3.4. Relationship between the Percentages of Positive Lymph Nodes and TRG following NAC and Surgery

We performed pathological analyses of lymph nodes using surgical specimens of the 62 patients and found a definitive correlation between TRG and postoperative pathological stage of the lymph nodes (Table 4). In the postoperative surgical specimens of the 28 patients in the TRG3 group, the number of positive lymph nodes was 152, and the rate of positive lymph nodes was 27.64%. In the postoperative specimens of the 28 patients in the TRG1+2 group, the number of positive lymph nodes was 131, and the rate of positive lymph nodes was 24.17%. In the postoperative specimens of the 6 patients in the TRG0 group, all lymph nodes were negative. These findings suggest that a lower pathological regression grade is associated with fewer positive lymph nodes, while a higher pathological regression grade is associated with a higher percentage of positive lymph nodes.

### 3.5. Correlation between Perioperative Imaging Findings and Postoperative Pathological Assessments

Patients fasted for more than 8 hours and underwent CT before and after NAC. CT was conducted using a 64-slice spiral CT scanner. Patients adopted a supine position, and tumor margins were identified for each slice at the arterial phase. Five circular regions of interest were selected from the slice with the longest in-plane diameter of the tumor and the abdominal aorta. CT values were recorded, and changes in CT values from before NAC to after surgery were calculated to analyze their relationships with TRG (Table 5). We found that changes in CT values were correlated with TRGs, and the extent of change was associated with patient prognosis. The TRG0 group showed the most significant change in CT values (81.77 ± 4.38). The change in CT values in the TRG1+2 group was approximately 69, and the least change in CT value was observed in the TRG3 group (65.67 ± 3.91).

## 4. Conclusions and Discussion

The use of NAC in advanced GC could downstage the tumor in most patients [17, 18]. However, since the assessment methods used before and after surgery differ and the preoperative imaging assessment is inaccurate for N staging, the TNM stage cannot truly reflect the downgraded tumor in patients. This study found that when only the T stage was considered, the difference in T stages between before and after surgery could accurately reflect the prognosis. Moreover, the follow-up findings showed that patients who received NAC had a more significant pathological regression, a high survival rate, and a favorable prognosis [19].

The percentage of positive lymph nodes is known to be a prognostic factor for metastasis, recurrence, and even survival of patients with cancer [19]. In this retrospective study, all 6 patients with complete pathological regression (TRG0) showed negative lymph nodes after receiving NAC. By comparison, the percentage of positive lymph nodes in the patients with partial regression (TRG1+2) was 24.17%, while that in the patients with little regression (TRG3) was 27.64%. The percentage of positive lymph nodes was higher in the patients with little regression than in those with completely or partially regressed tumors. This finding also indicates that NAC is effective in many patients. In addition, patients diagnosed with lymph node metastases after surgery had a poor prognosis, irrespective of the degree of tumor regression, but patients without lymph node metastases had a better prognosis [20].

We compared the findings of perioperative imaging assessments and postoperative pathological evaluations on remission by analyzing changes in CT values and TRGs. We demonstrated that the assessment results of the two methods were similar, but their results on complete remission were discordant. Moreover, we found that CT values were statistically different between the TRG0 and non-TRG0 groups, but not between the non-TRG0 groups; these findings might be due to the complexity of primary lesions. Lesions are generally composed of normal tissues, tumor-adjacent tissues, and cancerous tissues; in addition, necrotic tissues are occasionally present. These tissues represent a gradually evolving process of primary lesions, and they have slightly different CT values. Following NAC, the tumor may not completely regress, and necrosis may occur, thereby leading to the formation of scar tissues. The scar tissues interfere with CT, thus affecting the assessment of NAC effectiveness. Our study suggests that changes in CT values after chemotherapy could provide some insights into the tumor sensitivity to chemotherapy, thus allowing timely adjustment of the treatment plan. Moreover, CT is a noninvasive modality and can be performed before surgery; thus, this evaluation is more convenient in clinical practice. However, due to the diversified nature of tumor regressions to chemotherapy, some tumors may not show evident scarring after regression. Thus, the results would be more generalizable if further studies could stratify the patients based on the types of tissue changes following regression. In addition, biomarkers such as ERCC1 and ERCC2 were considered predictors of response following mFOLFOX-4 neoadjuvant chemotherapy [21]. Whether the combination of CT change and biomarker expression would be better predictors for prognosis would be examined as well. Finally, this was a small, single-center, retrospective study. Thus, further large-scale randomized controlled studies with a large sample size are needed to validate our present findings.

## Figures and Tables

**Table 1:** Basic characteristic of 62 patients.

	Patients (*n* = 62)	%
*Sex*		
Male	48	77.4
Female	14	22.6
Age (years; median, range)	56.5 (31-75)	
*Site of tumor*		
Cardia	2	3.2
Fundus of stomach	10	16.1
Gastric body	22	35.5
Antrum	25	40.3
Angle of stomach	3	4.9
*Degree of tissue differentiation*		
Poorly differentiated	43	69.4
Moderately differentiated	19	30.6
Well-differentiated	0	0

**Table 2:** Differences in TNM stages between before NAC and after surgery.

	0	IA	IB	IIA	IIB	IIIA	IIIB	IIIC	IV
Tumor stages before NAC	0	0	0	0	9	23	24	5	1
Tumor stages after NAC	6	1	2	11	6	13	7	15	1
*Z*	-0.21								
*p*	0.84								

**Table 3:** Differences in T stages between before NAC and after surgery.

	T0	T1	T2	T3	T4
T stages before NAC	0	0	0	8	54
T stages after NAC	6	2	10	11	33
*Z*	-3.47				
*p*	0.001				

**Table 4:** Relationships between percentages of positive lymph nodes and TRG following NAC and surgery.

Pathological assessments	Number of negative lymph nodes	Number of positive lymph nodes	Positive lymph nodes (%)
TRG0	111	0	0
TRG1+2	411	131	24.17
TRG3	398	152	27.64

**Table 5:** Comparison of imaging and pathological assessments of NAC effectiveness for GC.

Pathological assessment	Number of patients	Before NAC	After NAC	Change in CT value	*p* value^a^
TRG0	6	125.56 ± 21.69	43.22 ± 9.64	81.77 ± 4.38	
TRG1	14	123.40 ± 19.03	60.56 ± 8.66	69.18 ± 4.99	0.032
TRG2	14	119.89 ± 20.13	40.35 ± 10.56	69.44 ± 4.93	0.041
TRG3	28	127.26 ± 25.49	69.95 ± 13.88	65.67 ± 3.91	0.048

^a^Compared with TRG0.

## Data Availability

The datasets used or analyzed during this current study are available from the corresponding author on reasonable request.
